# The northernmost record of a blood-sucking ectoparasite, *Lipoptena fortisetosa* Maa (Diptera: Hippoboscidae), in Estonia

**DOI:** 10.3897/BDJ.7.e47857

**Published:** 2019-12-13

**Authors:** Olavi Kurina, Heli Kirik, Heino Õunap, Erki Õunap

**Affiliations:** 1 Institute of Agricultural and Environmental Sciences, Estonian University of Life Sciences, Kreutzwaldi st 5D, Tartu, Estonia Institute of Agricultural and Environmental Sciences, Estonian University of Life Sciences, Kreutzwaldi st 5D Tartu Estonia; 2 Estonian Environment Agency , Rõõmu tee 6, Tartu, Estonia Estonian Environment Agency , Rõõmu tee 6 Tartu Estonia; 3 Department of Zoology, Institute of Ecology and Earth Sciences, University of Tartu, Vanemuise 46, Tartu, Estonia Department of Zoology, Institute of Ecology and Earth Sciences, University of Tartu, Vanemuise 46 Tartu Estonia

**Keywords:** Diptera, DNA barcode, Hippoboscidae, deer keds, distribution, range expansion

## Abstract

**Background:**

Deer keds are obligatory haematophagous parasites of large homeothermic animals, particularly cervids. Two of the five known species occurring in Europe—*Lipoptena cervi* (Linnaeus) and *L. fortisetosa* Maa—are known to have a relatively wide distribution. *Lipoptena fortisetosa* is considered to have been introduced into Europe with sika deer from the Eastern Palaearctic and is continuously expanding its range. Little is known about the medical importance of deer keds, but they can cause hair loss in cervids and are suspected to be vectors of several diseases.

**New information:**

Details of the distribution of *Lipoptena fortisetosa* in Europe, including its northernmost record, are provided. This species has been shown to have a viable population in Southern Estonia. Furthermore, the differences from allied *L. cervi* are discussed, based on morphological and molecular characters.

## Introduction

**Figure 1.**
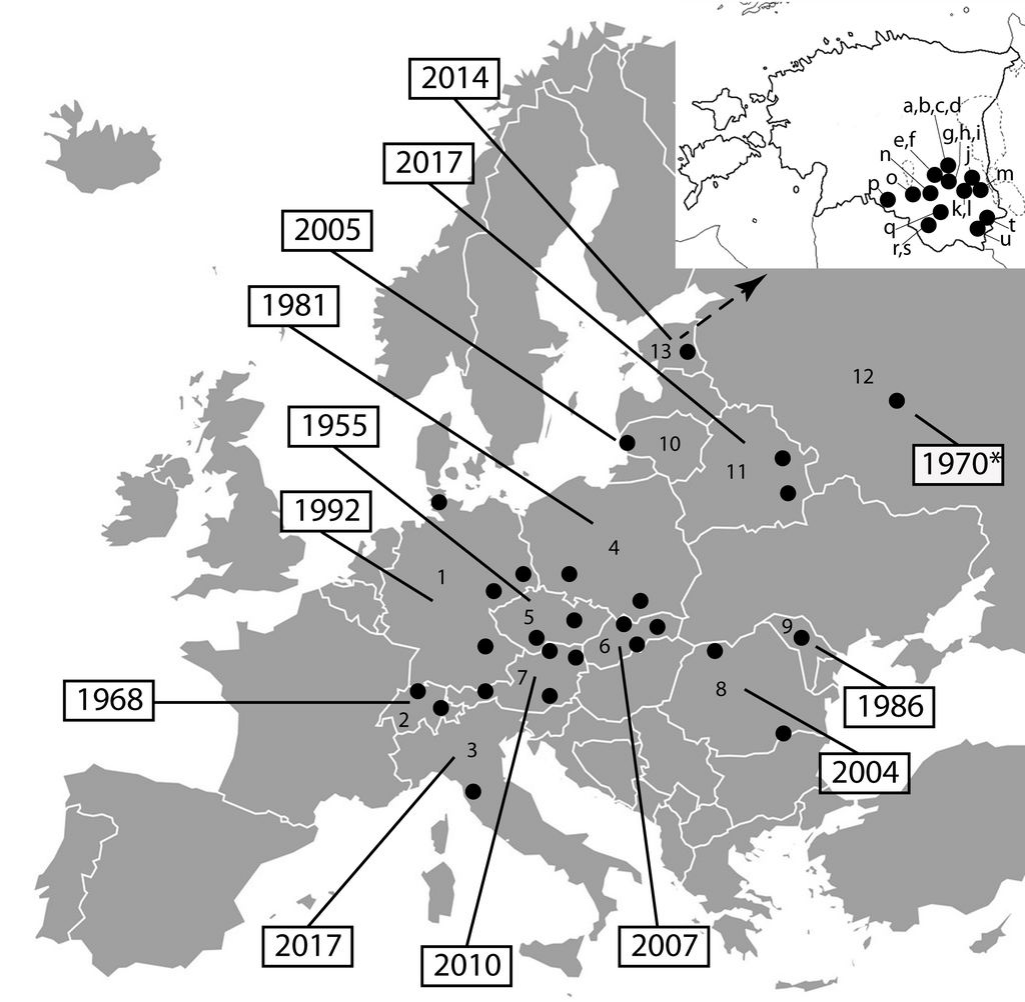
Distribution of *Lipoptena fortisetosa* in Europe, with year of the earliest known specimen for each particular country. Asterisk indicates a year of published reference without collected/observed specimen(s) data known. A dot on the map can represent several nearby records. The source references are: 1 – Germany (Schumann and Messner 1993, GBIF 2019), 2 – Switzerland (Büttiker 1994), 3 – Italy (Andreani et al. 2019), 4 – Poland (Borowiec and Zatwarnicki 1989, Kowal et al. 2009), 5 – Czech Republic (Theodor 1967, Sychra 2009), 6 – Slovak Republic (Oboňa et al. 2019), 7 – Austria (Schedl 2017, Schedl 2018), 8 – Romania (Pârvu 2005, Mihalca et al. 2019), 9 – Moldova (Metelitsa and Veselkin 1989), 10 – Lithuania (Dumčius and Pakalniškis 2005), 11 – Belarus (Ostrovsky 2017), 12 – Moscow district in Russia (Grunin 1970), 13 – Estonia (original data: letters correspond to the collecting localities in the Material section).

Deer keds (Insecta: Diptera: Hippoboscidae: *Lipoptena* spp.) parasitise wild as well as domestic animals, being most often associated with Cervidae and have been known to occasionally attack humans. Their medical impact is only superficially known, but as shown quite recently, deer keds are potential vectors of several diseases, for example, those caused by *Bartonella* spp., *Coxiella* spp. and *Rickettsia* spp. (Hornok et al. 2011, Lee et al. 2016, Szewczyk et al. 2017, Regier et al. 2018). Therefore, precise species identification, distribution details and bionomics are of utmost importance from the animal and human health perspective and imperative for vector control. There are 30 *Lipoptena* species known worldwide (Dick 2006), including five species recorded from Europe (Petersen 2013). Three of the five species have a restricted distribution in Southern Europe, including the Mediterranean islands, while *L. cervi* (Linnaeus, 1758) and *L. fortisetosa* Maa, 1965 have a more northern range (Petersen 2013). Having been described from Honshu Island in Japan (Maa 1965), *L. fortisetosa* has been subsequently widely reported from the Eastern Palaeractic (e.g. Dozanov 2003). In Europe, the species was first recorded from Czech Republic where it was initially described as a new species—*L. parvula* Theodore, 1967, but was later synonymised with *L. fortisetosa* by Grunin (1970). Thereafter, a number of records have been published from Central and Eastern European countries (see Fig. 1 for details). However, no records of *L. fortisetosa* westwards from Switzerland, northwards from the Moscow region in Russia or southwards from Tuscany in Italy were hitherto known.

During recent years, several specimens of deer keds, collected from Estonia and morphologically distinct from the common *L. cervi*, have come into the authors’ possession. Detailed investigation of these specimens, following the keys provided by Grunin (1970) and Büttiker (1994) and the differential diagnosis provided by Andreani et al. (2019), revealed them to be *L. fortisetosa*. This is the first time this species has been recorded from Estonia. Our study aims to investigate the morphological and molecular differences between *L. cervi* and *L. fortisetosa*, summarise the known distributions and provide the northernmost record of the latter.

## Materials and methods

Altogether, 35 specimens collected from 21 localities in South-eastern Estonia from 2014 to 2019 have been studied. The majority of the specimens were collected while they were attacking humans. The insects were caught manually and submerged in ethyl alcohol or pinned. This material is deposited in the insect collection of the Institute of Agricultural and Environmental Sciences, Estonian University of Life Sciences [former Institute of Zoology and Botany], Tartu, Estonia (IZBE), in the Zoological Museum University of Tartu, Estonia (TUZ) and in the private collection of Heino Õunap, Tartu, Estonia (PICHO). The habitus pictures have been stacked using the software LAS V.4.1.0. from several gradually focused images taken from pinned specimens by a Leica DFC 450 camera, attached to a stereomicroscope Leica 205C (for details, see Kurina et al. 2015).

Genomic DNA was extracted from one crushed hind leg of each specimen using High Pure PCR Template Preparation Kit (Roche Diagnostics GmbH, Mannheim, Germany). The extraction was carried out following the manufacturer’s instructions, with the exception that the first incubation step was 55ºC for two hours rather than one hour.

A 643-bp section from the 5' terminus of the mitochondrial cytochrome oxidase gene subunit I (COI), roughly corresponding to the standard DNA barcoding fraction of the gene (Hebert et al. 2004) was sequenced. PCR was performed in a total volume of 20 μl, with the reaction mixture containing 1X BD Advantage 2 PCR buffer, 1U BD Advantage 2 Polymerase mix (BD Biosciences, San Jose, USA), 0.2 mM dNTP (Thermo Scientific, Pittsburgh, USA), 5 pmol of primers LCO1490 (5'-ggtcaacaaatcataaagatattgg-3') and HC02198 (5'-taaacttcagggtgaccaaaaaatca-3') (Folmer et al. 1994) (replaced by MLepF1 (5’- GCTTTCCCACGAATAAATAATA-3’) (Hajibabaei et al. 2006) and LepR1 (5’-TAAACTTCTGGATGTCCAAAAAATCA-3’) (Hebert et al. 2004) for degraded samples) and 20–80 ng of purified genomic DNA. PCR was performed on a T1 thermocycler (Biometra, Göttingen, Germany) and the cycling parameters were: a 2-min denaturing step at 94°C, followed by 35 cycles of 30 s at 94°C, 30 s at 56°C (primers LCO1490 and HCO2198) or 51°C (primers MLepF1 and LepR1) and 60 s at 68°C with a subsequent 7-min final extension at 68°C. PCR products were visualised on a 1.6% agarose gel and 10 μl of the PCR solution was treated with FastAP thermosensitive alkaline phosphatase and exonuclease I (Thermo Scientific). One unit of both enzymes was added to the PCR solution, which was incubated for 15 min at 37°C, followed by 15 min inactivation at 80°C.

The DNA cycle sequencing was performed in a total volume of 10 μl using BigDye® Terminator v3.1 Cycle Sequencing Kit (Applied Biosystems, Foster City, CA). Cycling conditions were: 33 cycles of 20 s at 95°C, 20 s at 50°C (primers LCO1490 and HCO2198) or 47°C (primers MLepF1 and LepR1) and 60 s at 60°C. Both DNA strands were sequenced with 2 pmol of primers and sequences were resolved by 3730xl DNA Analyzer automated sequencer (Applied Biosystems) in Estonian Biocentre (Tartu, Estonia).

Consensus sequences were created using Geneious 7.1.9 (Biomatters Ltd, Auckland, New Zealand). Sequences were aligned using ClustalW (Thompson et al. 1994) implemented in BioEdit 7.0.5.2 (Hall 1999). Uncorrected pairwise genetic distances between the studied specimens were calculated using MEGA6 (Tamura et al. 2013).

In the Materials section of Taxon treatment below, only one specimen per collecting locality has been presented. For the full list of the studied specimens, including the comparative material of *L. cervi*, see Suppl. material 1.

## Taxon treatments

### Lipoptena fortisetosa

Maa, 1965

6E5FF2B2-4986-57D9-AD47-0A2B3DD7268A

#### Materials

- **Type status:** Other material. **Occurrence:** catalogNumber: IZBE0270001; recordedBy: T. Kesküla; individualCount: 1; sex: male; preparations: in ethyl alcohol; **Taxon:** scientificName: Lipoptena fortisetosa Maa, 1965; family: Hippoboscidae; genus: Lipoptena; specificEpithet: fortisetosa; scientificNameAuthorship: Maa, 1965; **Location:** country: Estonia; countryCode: EE; county: Tartu; municipality: Tartu city; locality: Tiksoja; decimalLatitude: 58.4147; decimalLongitude: 26.6380; **Identification:** identifiedBy: O. Kurina; dateIdentified: 2019; **Event:** samplingProtocol: captured while attacking humans; year: 2019; month: 7; day: 21; **Record Level:** type: Preserved specimen; institutionCode: EMY; collectionCode: IZBE; basisOfRecord: PreservedSpecimen
- **Type status:** Other material. **Occurrence:** catalogNumber: IZBE0270002; recordedBy: T. Kesküla; individualCount: 1; sex: female; preparations: in ethyl alcohol; **Taxon:** scientificName: Lipoptena fortisetosa Maa, 1965; family: Hippoboscidae; genus: Lipoptena; specificEpithet: fortisetosa; scientificNameAuthorship: Maa, 1965; **Location:** country: Estonia; countryCode: EE; county: Tartu; municipality: Tartu city; locality: Raudtee street; decimalLatitude: 58.3477; decimalLongitude: 26.6936; **Identification:** identifiedBy: O. Kurina; dateIdentified: 2019; **Event:** samplingProtocol: sweepnet; year: 2017; month: 8; day: 7; **Record Level:** type: Preserved specimen; institutionCode: EMY; collectionCode: IZBE; basisOfRecord: PreservedSpecimen
- **Type status:** Other material. **Occurrence:** catalogNumber: IZBE0270006; recordedBy: T. Kesküla; individualCount: 1; sex: male; preparations: in ethyl alcohol; **Taxon:** scientificName: Lipoptena fortisetosa Maa, 1965; family: Hippoboscidae; genus: Lipoptena; specificEpithet: fortisetosa; scientificNameAuthorship: Maa, 1965; **Location:** country: Estonia; countryCode: EE; county: Tartu; municipality: Kambja; locality: Variku forest; decimalLatitude: 58.3425; decimalLongitude: 26.6805; **Identification:** identifiedBy: O. Kurina; dateIdentified: 2019; **Event:** samplingProtocol: captured while attacking humans; year: 2019; month: 7; day: 4; **Record Level:** type: Preserved specimen; institutionCode: EMY; collectionCode: IZBE; basisOfRecord: PreservedSpecimen
- **Type status:** Other material. **Occurrence:** catalogNumber: IZBE0270007; recordedBy: M. Kruus; individualCount: 1; sex: male; preparations: in ethyl alcohol; **Taxon:** scientificName: Lipoptena fortisetosa Maa, 1965; order: Hippoboscidae; genus: Lipoptena; specificEpithet: fortisetosa; scientificNameAuthorship: Maa, 1965; **Location:** country: Estonia; countryCode: EE; county: Tartu; municipality: Kastre; locality: Haaslava; decimalLatitude: 58.3329; decimalLongitude: 26.8326; **Identification:** identifiedBy: O. Kurina; dateIdentified: 2019; **Event:** samplingProtocol: captured while attacking humans; year: 2019; month: 7; day: 31; **Record Level:** type: Preserved specimen; institutionCode: EMY; collectionCode: IZBE; basisOfRecord: PreservedSpecimen
- **Type status:** Other material. **Occurrence:** catalogNumber: PICHO07705; recordedBy: H. Õunap; individualCount: 1; sex: male; preparations: pinned; **Taxon:** scientificName: Lipoptena fortisetosa Maa, 1965; family: Hippoboscidae; genus: Lipoptena; specificEpithet: fortisetosa; scientificNameAuthorship: Maa, 1965; **Location:** country: Estonia; countryCode: EE; county: Tartu; municipality: Nõo; locality: Meeri; decimalLatitude: 58.2736; decimalLongitude: 26.4588; **Identification:** identifiedBy: H. Õunap; dateIdentified: 2016; **Event:** samplingProtocol: captured while attacking humans; year: 2016; month: 7; day: 15; **Record Level:** type: Preserved specimen; collectionCode: PICHO; basisOfRecord: PreservedSpecimen
- **Type status:** Other material. **Occurrence:** catalogNumber: IZBE0270008; recordedBy: K. Sammet; individualCount: 1; sex: male; preparations: in ethyl alcohol; **Taxon:** scientificName: Lipoptena fortisetosa Maa, 1965; family: Hippoboscidae; genus: Lipoptena; specificEpithet: fortisetosa; scientificNameAuthorship: Maa, 1965; **Location:** country: Estonia; countryCode: EE; county: Tartu; municipality: Nõo; locality: Peedu; decimalLatitude: 58.2060; decimalLongitude: 26.4303; **Identification:** identifiedBy: O. Kurina; dateIdentified: 2019; **Event:** samplingProtocol: captured while attacking humans; year: 2019; month: 7; day: 13; **Record Level:** type: Preserved specimen; institutionCode: EMY; collectionCode: IZBE; basisOfRecord: PreservedSpecimen
- **Type status:** Other material. **Occurrence:** catalogNumber: IZBE0270009; recordedBy: M. Kruus; individualCount: 1; sex: male; preparations: in ethyl alcohol; **Taxon:** scientificName: Lipoptena fortisetosa Maa, 1965; family: Hippoboscidae; genus: Lipoptena; specificEpithet: fortisetosa; scientificNameAuthorship: Maa, 1965; **Location:** country: Estonia; countryCode: EE; county: Tartu; municipality: Kastre; locality: Ignase; decimalLatitude: 58.2499; decimalLongitude: 26.8342; **Identification:** identifiedBy: O. Kurina; dateIdentified: 2019; **Event:** samplingProtocol: captured while attacking humans; year: 2019; month: 7; day: 23; **Record Level:** type: Preserved specimen; institutionCode: EMY; collectionCode: IZBE; basisOfRecord: PreservedSpecimen
- **Type status:** Other material. **Occurrence:** catalogNumber: PICHO07703; recordedBy: H. Õunap; individualCount: 1; sex: male; preparations: pinned; **Taxon:** scientificName: Lipoptena fortisetosa Maa, 1965; family: Hippoboscidae; genus: Lipoptena; specificEpithet: fortisetosa; scientificNameAuthorship: Maa, 1965; **Location:** country: Estonia; countryCode: EE; county: Tartu; municipality: Kambja; locality: Vana-Kuuste; decimalLatitude: 58.2680; decimalLongitude: 26.7866; **Identification:** identifiedBy: H. Õunap; dateIdentified: 2015; **Event:** samplingProtocol: captured while attacking humans; year: 2015; month: 7; day: 28; **Record Level:** type: Preserved specimen; collectionCode: PICHO; basisOfRecord: PreservedSpecimen
- **Type status:** Other material. **Occurrence:** catalogNumber: PICHO07707; recordedBy: H. Õunap; individualCount: 1; sex: female; preparations: pinned; **Taxon:** scientificName: Lipoptena fortisetosa Maa, 1965; family: Hippoboscidae; genus: Lipoptena; specificEpithet: fortisetosa; scientificNameAuthorship: Maa, 1965; **Location:** country: Estonia; countryCode: EE; county: Tartu; municipality: Kambja; locality: 3 km NO of Kambja; **Identification:** identifiedBy: H. Õunap; dateIdentified: 2016; **Event:** eventID: captured while attacking humans; year: 2016; month: 7; day: 30; **Record Level:** type: Preserved specimen; collectionCode: PICHO; basisOfRecord: PreservedSpecimen
- **Type status:** Other material. **Occurrence:** catalogNumber: IZBE0270013; recordedBy: K. Filippova; individualCount: 1; sex: female; preparations: in ethyl alcohol; **Taxon:** scientificName: Lipoptena fortisetosa Maa, 1965; family: Hippoboscidae; genus: Lipoptena; specificEpithet: fortisetosa; scientificNameAuthorship: Maa, 1965; **Location:** country: Estonia; countryCode: EE; county: Tartu; municipality: Kastre; locality: Järvselja; decimalLatitude: 58.2651; decimalLongitude: 27.3165; **Identification:** identifiedBy: O. Kurina; dateIdentified: 2019; **Event:** samplingProtocol: captured while attacking humans; year: 2019; month: 6; day: 27; **Record Level:** type: Preserved specimen; institutionCode: EMY; collectionCode: IZBE; basisOfRecord: PreservedSpecimen
- **Type status:** Other material. **Occurrence:** catalogNumber: IZBE0270015; recordedBy: L-M. Kurina; individualCount: 1; sex: male; preparations: in ethyl alcohol; **Taxon:** scientificName: Lipoptena fortisetosa Maa, 1965; family: Hippoboscidae; genus: Lipoptena; specificEpithet: fortisetosa; scientificNameAuthorship: Maa, 1965; **Location:** country: Estonia; countryCode: EE; county: Põlva; municipality: Põlva; locality: Mooste; decimalLatitude: 58.1679; decimalLongitude: 27.1423; **Identification:** identifiedBy: O. Kurina; dateIdentified: 2019; **Event:** samplingProtocol: captured while attacking humans; year: 2019; month: 7; day: 18; **Record Level:** type: Preserved specimen; institutionCode: EMY; collectionCode: IZBE; basisOfRecord: PreservedSpecimen
- **Type status:** Other material. **Occurrence:** catalogNumber: IZBE0270016; recordedBy: T. Tammaru; individualCount: 1; sex: male; preparations: in ethyl alcohol; **Taxon:** scientificName: Lipoptena fortisetosa Maa, 1965; family: Hippoboscidae; genus: Lipoptena; specificEpithet: fortisetosa; scientificNameAuthorship: Maa, 1965; **Location:** country: Estonia; countryCode: EE; county: Põlva; municipality: Põlva; locality: Karilatsi; decimalLatitude: 58.1293; decimalLongitude: 26.9031; **Identification:** identifiedBy: O. Kurina; dateIdentified: 2019; **Event:** samplingProtocol: captured while attacking humans; year: 2019; month: 7; day: 15; **Record Level:** type: Preserved specimen; institutionCode: EMY; collectionCode: IZBE; basisOfRecord: PreservedSpecimen
- **Type status:** Other material. **Occurrence:** catalogNumber: PICHO07702; recordedBy: E. Kaur; individualCount: 1; sex: male; preparations: pinned; **Taxon:** scientificName: Lipoptena fortisetosa Maa, 1965; family: Hippoboscidae; genus: Lipoptena; specificEpithet: fortisetosa; scientificNameAuthorship: Maa, 1965; **Location:** country: Estonia; countryCode: EE; county: Põlva; municipality: Räpina; locality: In bog close to Meelva Lake; decimalLatitude: 58.1200; decimalLongitude: 27.3708; **Identification:** identifiedBy: H. Õunap; dateIdentified: 2014; **Event:** samplingProtocol: captured while attacking humans; year: 2014; month: 9; day: 27; **Record Level:** type: Preserved specimen; collectionCode: PICHO; basisOfRecord: PreservedSpecimen
- **Type status:** Other material. **Occurrence:** catalogNumber: TUZ275550; recordedBy: M. Brotski; individualCount: 1; sex: female; preparations: pinned; **Taxon:** scientificName: Lipoptena fortisetosa Maa, 1965; family: Hippoboscidae; genus: Lipoptena; specificEpithet: fortisetosa; scientificNameAuthorship: Maa, 1965; **Location:** country: Estonia; countryCode: EE; county: Valga; municipality: Otepää; locality: Pühajärve; decimalLatitude: 58.0443; decimalLongitude: 26.4551; **Identification:** identifiedBy: O. Kurina; dateIdentified: 2019; **Event:** samplingProtocol: captured while attacking humans; year: 2019; month: 6; day: 12; **Record Level:** type: Preserved specimen; institutionCode: UTE; collectionCode: TUZ; basisOfRecord: PreservedSpecimen
- **Type status:** Other material. **Occurrence:** catalogNumber: PICHO07704; recordedBy: H. Õunap; individualCount: 1; sex: male; preparations: pinned; **Taxon:** scientificName: Lipoptena fortisetosa Maa, 1965; family: Hippoboscidae; genus: Lipoptena; specificEpithet: fortisetosa; scientificNameAuthorship: Maa, 1965; **Location:** country: Estonia; countryCode: EE; county: Valga; municipality: Otepää; locality: Purtsi; decimalLatitude: 58.0580; decimalLongitude: 26.1155; **Identification:** identifiedBy: H. Õunap; dateIdentified: 2016; **Event:** samplingProtocol: captured while attacking humans; year: 2016; month: 7; day: 15; **Record Level:** type: Preserved specimen; collectionCode: PICHO; basisOfRecord: PreservedSpecimen
- **Type status:** Other material. **Occurrence:** catalogNumber: IZBE0270018; recordedBy: H. Kirik; individualCount: 1; sex: female; preparations: in ethyl alcohol; **Taxon:** scientificName: Lipoptena fortisetosa Maa, 1965; family: Hippoboscidae; genus: Lipoptena; specificEpithet: fortisetosa; scientificNameAuthorship: Maa, 1965; **Location:** country: Estonia; countryCode: EE; county: Viljandi; municipality: Mulgi; locality: Lilli; decimalLatitude: 57.9744; decimalLongitude: 25.5562; **Identification:** identifiedBy: O. Kurina; dateIdentified: 2019; **Event:** samplingProtocol: captured while attacking humans; year: 2019; month: 7; day: 7; **Record Level:** type: Preserved specimen; institutionCode: EMY; collectionCode: IZBE; basisOfRecord: PreservedSpecimen
- **Type status:** Other material. **Occurrence:** catalogNumber: IZBE0270019; recordedBy: J. Ruusmaa; individualCount: 1; sex: female; preparations: in ethyl alcohol; **Taxon:** scientificName: Lipoptena fortisetosa Maa, 1965; family: Hippoboscidae; genus: Lipoptena; specificEpithet: fortisetosa; scientificNameAuthorship: Maa, 1965; **Location:** country: Estonia; countryCode: EE; county: Põlva; municipality: Kanepi; locality: South from Lake Uiakatsi; decimalLatitude: 57.9744; decimalLongitude: 25.5562; **Identification:** identifiedBy: O. Kurina; dateIdentified: 2019; **Event:** samplingProtocol: captured while attacking humans; year: 2019; month: 7; day: 16; **Record Level:** type: Preserved specimen; institutionCode: EMY; collectionCode: IZBE; basisOfRecord: PreservedSpecimen
- **Type status:** Other material. **Occurrence:** catalogNumber: PICHO07701; recordedBy: E. Kaur; individualCount: 1; sex: female; preparations: pinned; **Taxon:** scientificName: Lipoptena fortisetosa Maa, 1965; family: Hippoboscidae; genus: Lipoptena; specificEpithet: fortisetosa; scientificNameAuthorship: Maa, 1965; **Location:** country: Estonia; countryCode: EE; county: Valga; municipality: Otepää; locality: Lossiküla; decimalLatitude: 57.9025; decimalLongitude: 26.2747; **Identification:** identifiedBy: H. Õunap; dateIdentified: 2014; **Event:** samplingProtocol: captured while attacking humans; year: 2014; month: 6; day: 28; **Record Level:** type: Preserved specimen; collectionCode: PICHO; basisOfRecord: PreservedSpecimen
- **Type status:** Other material. **Occurrence:** catalogNumber: IZBE0270021; recordedBy: L-M. Kurina; individualCount: 1; sex: female; preparations: in ethyl alcohol; **Taxon:** scientificName: Lipoptena fortisetosa Maa, 1965; family: Hippoboscidae; genus: Lipoptena; specificEpithet: fortisetosa; scientificNameAuthorship: Maa, 1965; **Location:** country: Estonia; countryCode: EE; county: Valga; municipality: Valga; locality: Lüllemäe; decimalLatitude: 57.7514; decimalLongitude: 26.3765; **Identification:** identifiedBy: O. Kurina; dateIdentified: 2019; **Event:** samplingProtocol: captured while attacking humans; year: 2019; month: 7; day: 15; **Record Level:** type: Preserved specimen; institutionCode: EMY; collectionCode: IZBE; basisOfRecord: PreservedSpecimen
- **Type status:** Other material. **Occurrence:** catalogNumber: IZBE0270022; recordedBy: E. Õunap; individualCount: 1; sex: male; preparations: pinned; **Taxon:** scientificName: Lipoptena fortisetosa Maa, 1965; family: Hippoboscidae; genus: Lipoptena; specificEpithet: fortisetosa; scientificNameAuthorship: Maa, 1965; **Location:** country: Estonia; countryCode: EE; county: Võru; municipality: Võru; locality: Piusa; decimalLatitude: 57.8400; decimalLongitude: 27.4702; **Identification:** identifiedBy: O. Kurina; dateIdentified: 2019; **Event:** samplingProtocol: captured while attacking humans; year: 2019; month: 7; day: 13; **Record Level:** type: Preserved specimen; institutionCode: EMY; collectionCode: IZBE; basisOfRecord: PreservedSpecimen
- **Type status:** Other material. **Occurrence:** catalogNumber: IZBE0270024; recordedBy: E. Õunap; individualCount: 1; sex: female; preparations: pinned; **Taxon:** scientificName: Lipoptena fortisetosa Maa, 1965; family: Hippoboscidae; genus: Lipoptena; specificEpithet: fortisetosa; scientificNameAuthorship: Maa, 1965; **Location:** country: Estonia; countryCode: EE; county: Võru; municipality: Võru; locality: Vana-Vastseliina; decimalLatitude: 57.7338; decimalLongitude: 27.3572; **Identification:** identifiedBy: O. Kurina; dateIdentified: 2019; **Event:** samplingProtocol: captured while attacking humans; year: 2019; month: 7; day: 15; **Record Level:** type: Preserved specimen; institutionCode: EMY; collectionCode: IZBE; basisOfRecord: PreservedSpecimen

#### Diagnosis

**Figure 2.**
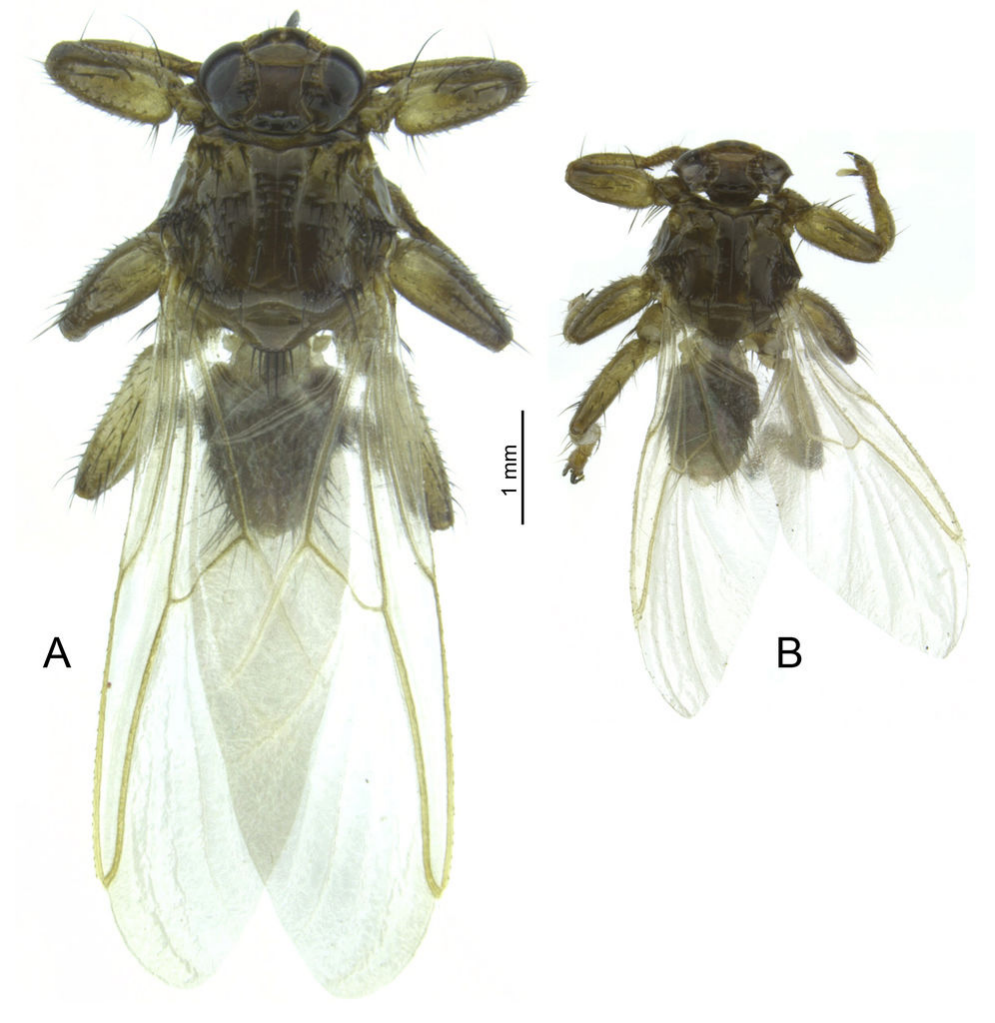
Habitus of females of *Lipoptena cervi* (A) and *L. fortisetosa* (B).

**Figure 3.**
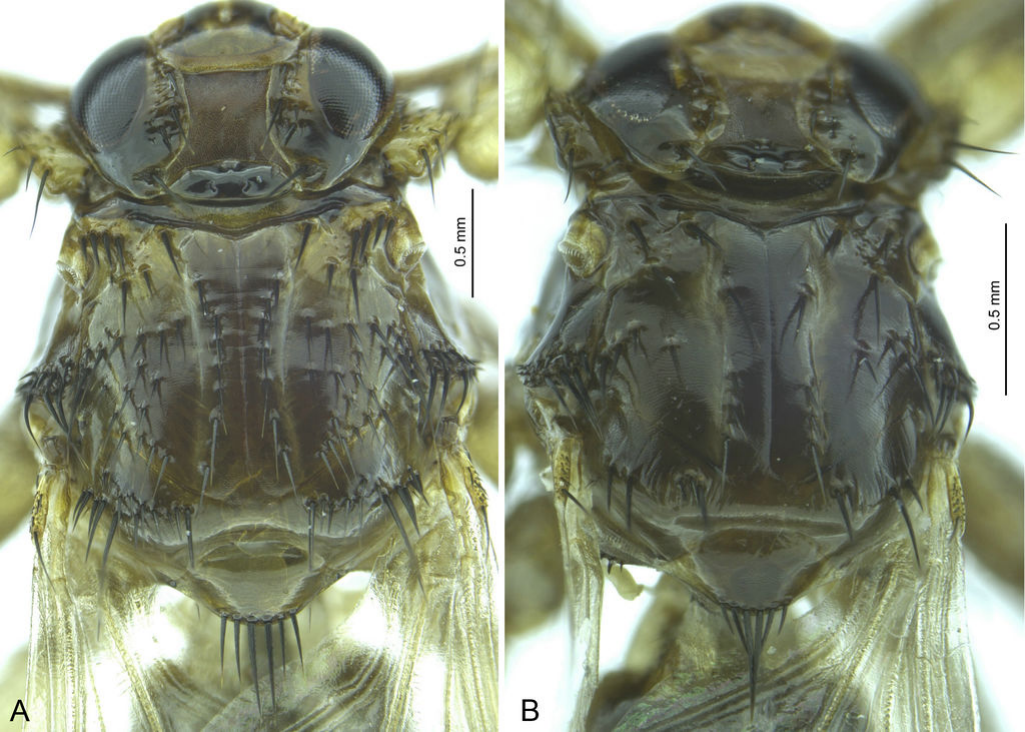
Dorsal view of the female thorax of *Lipoptena cervi* (A) and *L. fortisetosa* (B).

The imago of *L. fortisetosa* is considerably smaller than that of *L. cervi* (Fig. 2) and the chaetotaxy of the thorax differs between the two (Fig. 3). In particular, the setae on the scutum of *L. cervi* are more numerous and variable in size than those in *L. fortisetosa*, these including 3–4 strong setae above the thoracic spiracle which are absent in *L. fortisetosa*. In addition, the head of *L. fortisetosa* is rhomboid (ovoid in *L. cervi*) and the abdomen is less sclerotised and lighter than that of *L. cervi*. The female of *L. fortisetosa* has one discernible pregenital sclerite, while there are three pregenital aligned sclerites in *L. cervi*. The male of *L. fortisetosa* has the aedeagus apically bilobate while it is conical in *L. cervi* (see also Andreani et al. 2019 for details).

The identities of the fragments of the COI gene obtained in this study were double-checked by BLAST search in GenBank. One hundred percent identity with sequences stored in the GenBank was recovered for several specimens of both *L. fortisetosa* and *L. cervi*, corroborating the correctness of our morphological identification.

To place our results in a wider context, original COI sequences of *Lipoptena* spp. were analysed, together with the COI data of their conspecifics available in the NCBI GenBank (accessed 03 October 2019). In total, our data matrix comprised 63 sequences of *L. cervi* (7 original, 56 downloaded) and 10 sequences of *L. fortisetosa* (7 original, 3 downloaded) (Suppl. material 2). Intraspecific pairwise genetic distances varied between 0.000 and 0.026 (average 0.002 ± 0.004 SD) in *L. cervi*, and 0.000 and 0.023 (average 0.01 ± 0.007 SD) in *L. fortisetosa*, respectively. Interspecific pairwise genetic distances were, however, significantly larger: from 0.065 to 0.086 (average 0.075 ± 0.004 SD). Reliable identification of *L. cervi* and *L. fortisetosa* is, therefore, possible using both morphological and molecular methods.

#### Biology

In the Eastern Palaearctic, *L. fortisetosa* is described as a common parasite on sika deer (Fukumoto et al. 2000, Nakayama 2007), but it has also been found to attack *Capreolus* species (Choi et al. 2013) and even passerine birds (Yamauchi et al. 2009). In Europe, *L. fortisetosa* has been observed to attack deer, cattle, goats, sheep and dogs, but also humans (Metelitsa and Veselkin 1989, Büttiker 1994, Schedl 2018, Mihalca et al. 2019). In Estonia, this species has often been observed to attack humans, which corroborates recent data from Slovakia (Oboňa et al. 2019). *L. fortisetosa* is thought to be a multivoltine species in Europe, with adults appearing from June to October, while the adults of sympatric but univoltine *L. cervi* are present from August to October (Kowal et al. 2016). Like other deer keds, adults of *L. fortisetosa* lose their wings after finding an acceptable host and start sucking its blood. Afterwards, the females give birth to full-grown larvae which forego feeding, leave the host shortly after birth and pupate immediately after falling to the ground. Newly hatched adults of the next generation start looking for a mammalian host soon after eclosion (e.g. Hutson 1984, Regier et al. 2018).

## Discussion

The first record of *L. fortisetosa* in Europe dates back more than 60 years when the species was collected in Czech Republic (Theodor 1967). Since then, its range has remarkably expanded (see Fig. 1 for details). The species is assumed to have Eastern Palaearctic origin and emerged in Europe probably together with the sika deer (Mihalca et al. 2019), which has been introduced to Europe repeatedly during the last 150 years. To date, sika deer has established itself in the wild and is considered an invasive species in several European countries (Bartoš 2009). As *L. fortisetosa* is adapted to parasitise a wide range of homeothermic animals (see above), there are no obvious limitations to its further expansion. During the last years, northward range expansion has been observed (Fig. 1) and, by now, *L. fortisetosa* has probably established a viable population in Southern Estonia. Very few sika deer individuals have been observed in Estonia during the last decades (Käärt 2014, T. Randveer pers. comm.). Therefore, these animals could not have served as hosts to the *L. fortisetosa* specimens collected from 21 remote localities in the south-eastern part of the country during the last five years. The host species of *L. fortisetosa* in Estonia remain thus unknown, but red deer (*Cervus elaphus* L.), roe deer (*Capreolus* capreolus (L.)) and moose (*Alces alces* (L.)) are the most likely candidates. It cannot be completely ruled out that such a range expansion could also be a response to global climate change. In the case of the congener, *L. cervi*, the range expansion northwards in Fennoscandia during last decades is argued to be driven by the host density changes and migration (mainly the moose), as well as climatic factors (overview by Välimäki et al. 2010).

All Estonian specimens of *L. fortisetosa* but two were collected from June to the beginning of August, while the comparative material of *L. cervi* was collected from mid-August to September (Suppl. material 1). However, one *L. fortisetosa* specimen was collected in late August and another one in late September. In Central Europe, there are records of *L. fortisetosa* from September and October, which means that some adults of this species are active simultaneously with *L. cervi* (Büttiker 1994, Oboňa et al. 2019).

Jaakola et al. (2015) reported that populations of *L. cervi* in Fennoscandia are genetically rather homogenous. Our results generally support their conclusion, as five out of the seven sequenced Estonian specimens had COI haplotypes identical to those of the Scandinavian ones (but see Suppl. material 2).

The *L. fortisetosa* COI sequences available in the GenBank hinted that this species is genetically diverse and has geographically distinct lineages in Europe, as the uncorrected genetic distance between specimens collected from Lithuania (Paulauskas et al. 2017) and Romania (Mihalca et al. 2019) is as high as 0.023 (Suppl. material 2). Quite surprisingly, our study revealed that both Lithuanian and Romanian haplotypes of *L. fortisetosa* are present in Estonia, thus rejecting the hypothesis of geographically distinct lineages (Suppl. material 2). The importance of such a lack of phylogeographic pattern is currently unclear. On the one hand, it may indicate that the population of *L. fortisetosa* that acted as a source of introduction to Europe was itself genetically diverse. The other possibility is that *L. fortisetosa* has been introduced to Europe from several genetically distinct source populations. In that case, its genetic diversity in Estonia has been caused by the range expansion of several introduced lineages that have ultimately become sympatric in Europe. Genetic studies of the Asian populations of *L. fortisetosa* are required for further insight.

## Supplementary Material

7FDDE5E5-5246-502A-8479-7DD34FDE8859 10.3897/BDJ.7.e47857.suppl1 Supplementary material 1 Full list with details of studied Lipoptena fortisetosa and L. cervi specimens. GenBank codes are provided for sequenced specimens. Abbreviations: m = males, f = females Data type: Studied specimens data
File: oo_363764.xls https://binary.pensoft.net/file/363764 Kurina, O., Kirik, H., Õunap, H., Õunap, E.

C458A0A6-6824-57C2-B290-0DFF3F962300 10.3897/BDJ.7.e47857.suppl2 Supplementary material 2 Uncorrected pairwise genetic distances between the partial COI sequences of Lipoptena cervi and L. fortisetosa. Interspecific genetic distances are highlighted by bold type. Original sequences created during this study are indicated with an asterisk (*) Data type: Genomic data
File: oo_363765.xls https://binary.pensoft.net/file/363765 Kurina, O., Kirik, H., Õunap, H., Õunap, E.

XML Treatment for Lipoptena fortisetosa
